# Transient elastography and serum markers of liver fibrosis associate with epicardial adipose tissue and coronary artery calcium in NAFLD

**DOI:** 10.1038/s41598-022-10487-3

**Published:** 2022-04-21

**Authors:** Carolina M. Perdomo, Ana Ezponda, Jorge M. Núñez-Córdoba, José I. Herrero, Gorka Bastarrika, Gema Frühbeck, Javier Escalada

**Affiliations:** 1 Department of Endocrinology and Nutrition, Clínica Universidad de Navarra, Pio XII, 36, 31008 Pamplona, Spain; 2 Department of Radiology, Clínica Universidad de Navarra, Pamplona, Spain; 3 Research Support Service, Central Clinical Trials Unit, Clínica Universidad de Navarra, Pamplona, Spain; 4 Hepatology Unit, Clínica Universidad de Navarra, Pamplona, Spain; 5 CIBERehd (CIBER Enfermedades Hepáticas y Digestivas), Madrid, Spain; 6 CIBERObn (CIBER Fisiopatología de la Obesidad y Nutrición), Instituto de Salud Carlos III, Madrid, Spain; 7 Present Address: IdiSNA (Instituto de Investigación en la Salud de Navarra), Pamplona, Spain

**Keywords:** Cardiology, Endocrinology, Gastroenterology, Risk factors

## Abstract

Non-alcoholic fatty liver disease (NAFLD) is associated with cardiovascular disease morbimortality. However, it is not clear if NAFLD staging may help identify early or subclinical markers of cardiovascular disease. We aimed to evaluate the association of liver stiffness and serum markers of liver fibrosis with epicardial adipose tissue (EAT) and coronary artery calcium (CAC) in an observational cross-sectional study of 49 NAFLD patients that were seen at Clínica Universidad de Navarra (Spain) between 2009 and 2019. Liver elastography and non-invasive fibrosis markers were used to non-invasively measure fibrosis. EAT and CAC, measured through visual assessment, were determined by computed tomography. Liver stiffness showed a direct association with EAT (r = 0.283, p-value = 0.049) and CAC (r = 0.337, p-value = 0.018). NAFLD fibrosis score was associated with EAT (r = 0.329, p-value = 0.021) and CAC (r = 0.387, p-value = 0.006). The association of liver stiffness with CAC remained significant after adjusting for metabolic syndrome features (including carbohydrate intolerance/diabetes, hypertension, dyslipidaemia, visceral adipose tissue, and obesity). The evaluation of NAFLD severity through liver elastography or non-invasive liver fibrosis biomarkers may contribute to guide risk factor modification to reduce cardiovascular risk in asymptomatic patients. Inversely, subclinical cardiovascular disease assessment, through Visual Scale for CAC scoring, may be a simple and effective measure for patients with potential liver fibrosis, independently of the existence of other cardiovascular risk factors.

## Introduction

Non-alcoholic fatty liver disease (NAFLD) is a highly prevalent disease worldwide that has become a challenge for health services due to its known relation to cardiovascular and liver morbimortality^1^. The presence and severity of fibrosis is the determining factor associated with higher mortality from any cause, although mainly due to cardiovascular disease (CVD)^2^. NAFLD is now considered an additional independent risk factor for CVD^3,3^, and its relation with subclinical cardiac damage has already been evidenced^5^. The association of NAFLD and CVD is complex; insulin resistance, proinflammatory cytokines, lipotoxicity, oxidative stress and endothelial dysfunction may all contribute to the development and progression of CVD^6,6^.

Coronary artery calcium (CAC)^8,8^ and epicardial adipose tissue (EAT)^10,10^ are considered surrogate markers of coronary artery disease and have significantly improved the cardiovascular risk classification in asymptomatic individuals^8–11^. EAT is a novel clinical biomarker not only associated with accelerated progression of subclinical coronary atherosclerosis^10,10^, but also mediator of cardiac arrhythmias^12^, left ventricular diastolic dysfunction^13^, and stroke^14^. EAT has important physiological functions^15^, nonetheless, excessive EAT leads to a proinflammatory state with adverse effects on the myocardium.

Liver biopsy is the reference method for assessing the severity of NAFLD, however, it is an invasive non-cost-effective procedure for a highly prevalent disease. Liver elastography (LE)^16^ and non-invasive fibrosis markers (e.g. NAFLD Fibrosis Score [NFS]^17^ and Fibrosis 4 Score [FIB-4]^18^) have shown high diagnostic precision for advance stages of liver fibrosis (F3–F4). Due to the high prevalence of NAFLD, it is of interest to investigate which patient is at a higher risk for cardiovascular abnormalities. The aim of this study was to assess the association of NAFLD severity, specifically through LE and non-invasive markers of liver fibrosis, with EAT and CAC.

## Materials and methods

### Study patients

This was a retrospective study of adult patients with NAFLD that were seen at Clínica Universidad de Navarra (Spain) between 2009 and 2019. The research ethics committee of the University of Navarra approved the study protocol (2019.080). Informed consent was obtained from all subjects involved in the study. Eligible patients were those that had LE, whole body scan computed tomography (CT-WBS) or computed tomography thoracic scan (CT-TS), and blood test at the same visit. In our Centre, CT-WBS/CT-TS and laboratory tests are routinely performed on the same day (or within a few days) of the initial visit. Epidemiological, clinical, laboratory and radiological information were obtained from patient records.

**Figure 1:**
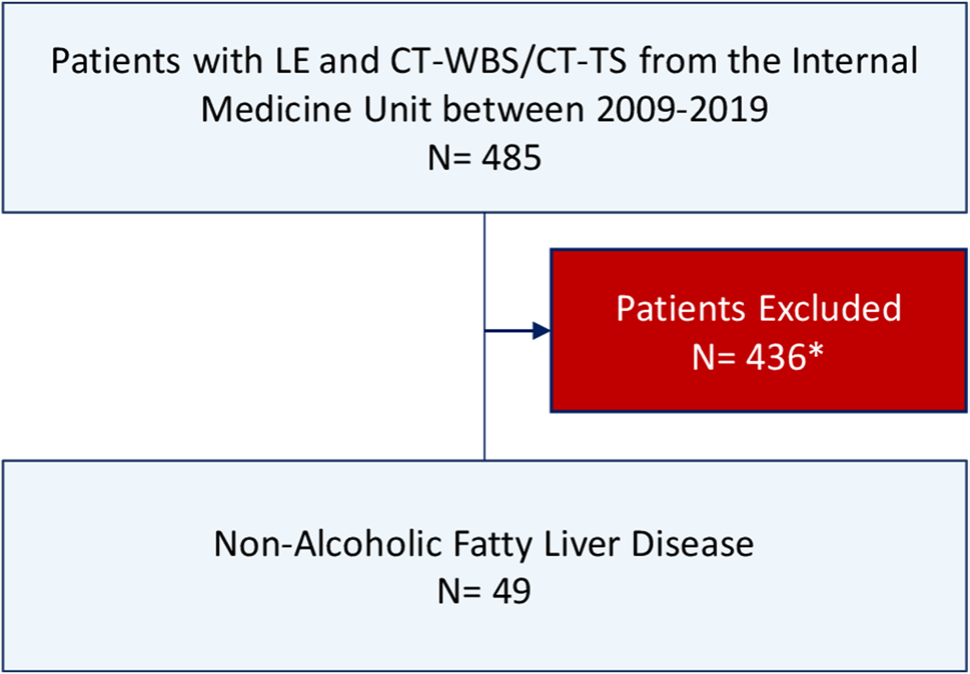
*LE* liver elastography, *CT-WBS* computed tomography whole body scan, *CT-TS* computed tomography thoracic scan. *Of the initial cohort of 485 patients, 436 were excluded for one or more of the following criteria that may have affected cardiovascular outcomes or had a different liver disease: personal history of cardiovascular disease (n = 12); active malignancy (n = 52); endocrine diseases (n = 1); excessive alcohol consumption (n = 125); viral hepatitis (n = 123); autoimmune liver disease (n = 28); toxic hepatitis (n = 10); steatogenic drugs (n = 2); iron overload (n = 8); alfa1-antitripsin deficit (n = 6); cirrhosis (n = 7); inflammatory diseases (n = 10); portal hypertension (n = 12) and other liver disease (cholestasis, cystic fibrosis, amyloidosis, Gilbert’s syndrome, paludism, thalassemia) (n = 40).

All subjects were non-symptomatic with no history of malignancy. Alcohol consumption was specifically investigated by interviewing the patients. Exclusion criteria included previous liver disease of other etiologies, such as alcoholic fatty liver disease, autoimmune or viral hepatitis, drug-induced fatty liver disease (e.g. amiodarone, systemic corticosteroids, valproic acid, carbamazepine, antiretroviral drugs, methotrexate or tamoxifen), autoimmune hepatitis, cholestatic liver disease, genetic liver disease (e.g. haemochromatosis), endocrinological disorders (e.g. hypopituitarism, hypothyroidism), inborn errors of metabolism or nutritional disorders (e.g. starvation, parenteral nutrition), systematic inflammatory disease and malignant disease. Additionally, patients with a personal history of cerebral vascular diseases (including transient ischemic attack), ischemic heart diseases, heart failure, atrial fibrillation, pericarditis, valvular disease or any other heart disease prior to index date were excluded (Fig. 1). All patients had a negative history of alcohol abuse as indicated by a daily ethanol consumption of less than 20 g in women, and less than 30 g in men.

### Non-invasive evaluation suggestive of fibrosis

We measured the liver stiffness using transient LE (Fibroscan^®^; Echosens, Paris, France)^16^. An experienced technician performed LE measurements. Only valid measurements were included. We used NFS^17^ and FIB-4 Score^18^ as non-invasive fibrosis serum markers. In NAFLD, a LE < 8.2 kPa is considered as low likelihood of fibrosis and ≥ 8.2 kPa is considered as clinically significant fibrosis (liver biopsy: ≥ F2); a NFS < − 1.455 is an approximate F0–F2 histological fibrosis stage, between ≥ − 1.455 and ≤ 0.676 is an indeterminant score, > 0.676 is an approximate F3–F4 histological fibrosis stage. On the other hand, a FIB-4 Score < 1.30 is an approximate F0–F1 histological fibrosis stage, between ≥ 1.30 and ≤ 2.67 is an indeterminant score, > 2.67 is an approximate F3–F4 histological fibrosis stage.

### Whole‑body scan computed tomography protocol

All examinations were performed using a sixty-four-row multidetector CT (64-MDCT) (SOMATOM Definition and SOMATOM Sensation-64, Siemens Healthcare, Forchheim, Germany), with tube voltage and reference tube current time product variable depending on region included. All images were stored in picture archiving and communication system (PACS). The protocol of CT-WBS included: low-dose chest CT (120 kV and 40 mA/s) without contrast material, CAC measurement (120 kV and 138 mA/s) in cases where coronary calcification was identified in low-dose chest CT, abdominopelvic CT (120 kV and 180 mA/s) performed after intravenous injection of 120-mL iodinated contrast medium (Omnipaque TM 300 (iohexol), 300 mg I/mL, GE Healthcare Bio-Sciences, Madrid, Spain), at 2 mL/s. Portal phase was acquired at 65 s.

From January 2020 to May 2020, CT-WBS images were reobtained from PACS to measure EAT, visual scoring of CAC, subcutaneous adipose tissue (SCAT) and visceral adipose tissue (VAT) by a specialized reader. EAT was defined as all adipose tissue enclosed by the visceral pericardium, including all fat directly surrounding the coronary arteries. The radiologist examined the CT-WBS and semiautomatically quantified EAT including voxels with attenuation values between − 45 to − 190 Hounsfield units. Visual evaluation of CAC is feasible and correlates well with the Agatston score showing a good inter-reader evaluation^19^. We used the ordinal CAC scoring for visual assessment of CAC^20^. The images were reviewed to evaluate the linear extent of calcium along the four main coronary arteries (right, circumflex, left main and left anterior descending). Calcification was scored as follows: 0 (no visible calcification); 1 (mild calcification; less than one-third of the length of the artery showed calcification); 2 (moderate calcification; one-third to two-thirds of the artery showed calcification); 3 (severe calcification; more than two-thirds of the artery showed calcification). Each patient received a CAC score ranging from 0 to 12 (0: very low risk; 1–3: mild to moderately increased risk; 4–12: moderately to severely increased risk). VAT and SCAT measurement were quantified semi-automatically including voxels with attenuation values between − 45 to − 190 Hounsfield units. The abdominal muscular wall was traced manually to separate VAT from the SCAT. The VAT/SCAT ratio was also measured due to its known correlation to cardiovascular risk, beyond body mass index (BMI) and VAT^21^.

### Statistical analysis

Demographic and clinical characteristics of patients were summarized using mean and standard deviation (SD), median, percentiles 25 (p25) and 75 (p75), and percentages. Correlations were evaluated with the estimation of the product-moment correlation coefficient (r). Multivariable linear regression models were used to assess the relationship between EAT/CAC and the severity of NAFLD, adjusting for potential confounders, including sex, obstructive sleep apnoea syndrome, hyperuricemia, statin therapy, aspirin therapy, smoking, and metabolic syndrome features, such as carbohydrate intolerance, diabetes, hypertension, dyslipidaemia, obesity, VAT, and VAT/SCAT ratio. All analyses were performed with Stata 14 (StataCorp. 2015. Stata Statistical Software: Release 14. College Station, TX: StataCorp LP). p < 0.05 was considered statistically significant.

### Institutional Review Board statement

This study was conducted in accordance with the Declaration of Helsinki. The study protocol was approved by the Research Ethics Committee of Universidad de Navarra (protocol code 2019.080; 7th of June 2019).

## Results

A total of 49 NAFLD patients were included in the analyses. Table 1 displays main demographic and clinical characteristics of patients. Mean age was 58 years (SD: 11.28; range: 18–83). Most patients were men (81.6%) and overweight or obese (85.7%). Impaired fasting glucose or diabetes was diagnosed in 59.2% of patients. Mean EAT was 177.7 cm^3^ (SD: 95.03, p25: 116.87, p75: 222.30). Median CAC score was 1 (p25: 0, p75: 3, range: 0–9). EAT and CAC were directly correlated (r = 0.429; p = 0.002).

**Table 1:** Characteristics of non-alcoholic fatty liver disease patients (n = 49).

Characteristics	Total
Age, y	58 (11.28)
Men, n (%)	40 (81.63)
Waist circumference, cm	105 (14.01)
Body mass index, kg/m^2^	30.1 (5.5)
Overweight, n (%)	21 (42.86)
**Obesity, n (%)**	
Class 1	14 (28.57)
Class 2	4 (8.16)
Class 3	3 (6.12)
Total body fat (CUNBAE), %	33.79 (8.15)
VAT, mL	4558.57 (2113.57)
SCAT, mL	6905.98 (3873.47)
VAT/SCAT ratio	0.74 (0.41)
Hypertension, n (%)	19 (38.78)
Impaired fasting glucose, n (%)	18 (36.73)
Diabetes, n (%)	11 (22.45)
Dyslipidaemia, n (%)	24 (48.98)
OSA, n (%)	7 (14.30)
**Smoking, n (%)**	
Current	10 (20.41)
Former	22 (44.90)
Never	17 (34.69)
CVD family history, n (%)	10 (20.41)
Antihypertensive therapy, n (%)	16 (32.70)
**Glucose lowering therapy, n (%)**	8 (16.30)
Metformin	5 (10.20)
Glinides	1 (2.00)
SGLT2 inhibitors	3 (6.10)
DPP4 inhibitors	3 (6.10)
Statin therapy, n (%)	13 (26.50)
Aspirin therapy, n (%)	3 (6.10)
Hypouricemic therapy, n (%)	4 (8.20)
Glucose, mg/dL	111 (30)
HbA1c, %	6.0 (1.0)
Insulin, U/mL	16.24 (8.86)
HOMA-IR	1.36 (2.50)
Triacylglycerol, mg/dL	127 (55)
Total cholesterol, mg/dL	188 (44)
LDL cholesterol, mg/dL	120 (57)
HDL cholesterol, mg/dL	50 (15)
ALT, IU/L	36 (23.40)
AST, IU/L	23 (11.57)
AST/ALT	0.7 (0.25)
ALP, IU/L, median (p25, p75)	47 (28, 74)
GGT, IU/L	64 (33.66)
Platelet count, × 10^3^/µL	228 (62.97)
Albumin, g/dL	4.78 (0.32)
Urate, mg/dL	11.2 (15.3)
Creatinine, mg/dL	3.9 (13.8)
Urine albumin to creatinine ratio, mg/g	4.9 (2.2)

Values are expressed as mean (SD), unless otherwise stated.

*ALT* Alanine transaminase, *ALP* alkaline phosphatase, *AST* aspartate transaminase, *BMI* body mass index, *CUNBAE* Clínica Universidad de Navarra-Body Adiposity Estimator, *CVD* cardiovascular disease. *DPP4* dipeptidyl peptidase 4, *GGT* gamma-glutamyl transpeptidase, *HbA1c* hemoglobin A1c, *HOMA-IR* homeostatic model assessment of insulin resistance, *OSA* obstructive sleep apnea, *SCAT* subcutaneous adipose tissue, *SGLT2* sodium-glucose co-transporter-2, *VAT* visceral adipose tissue.

Mean liver stiffness was 6.28 kPa (SD: 3.06, p25: 4.30, p75: 7.30, liver stiffness ≥ 8.2 kPa: 16.3% of patients). Mean FIB-4 Score was 1.08 (SD: 0.48, p25: 0.72, p75: 1.40). Mean NFS was − 1.45 (SD: 1.21, p25: − 2.44; p75: − 0.68). According to FIB-4 Score, 73.5% of the patients were classified as with mild fibrosis or absence of significant fibrosis (FIB-4 Score < 1.30) and 26.53% of the patients were classified as with an indeterminant range fibrosis status (FIB-4 Score ≥ 1.30 and ≤ 2.67). No patient had a FIB-4 Score above 2.67. According to NFS, 46.9% of patients had absence of significant liver fibrosis (NFS ≤ 1.455), 49.0% were classified as with an indeterminant range fibrosis status (NFS: ≥  − 1.455 and ≤ 0.676) and 4.1% had presence of significant liver fibrosis (NFS: > 0.676).

**Figure 2:**
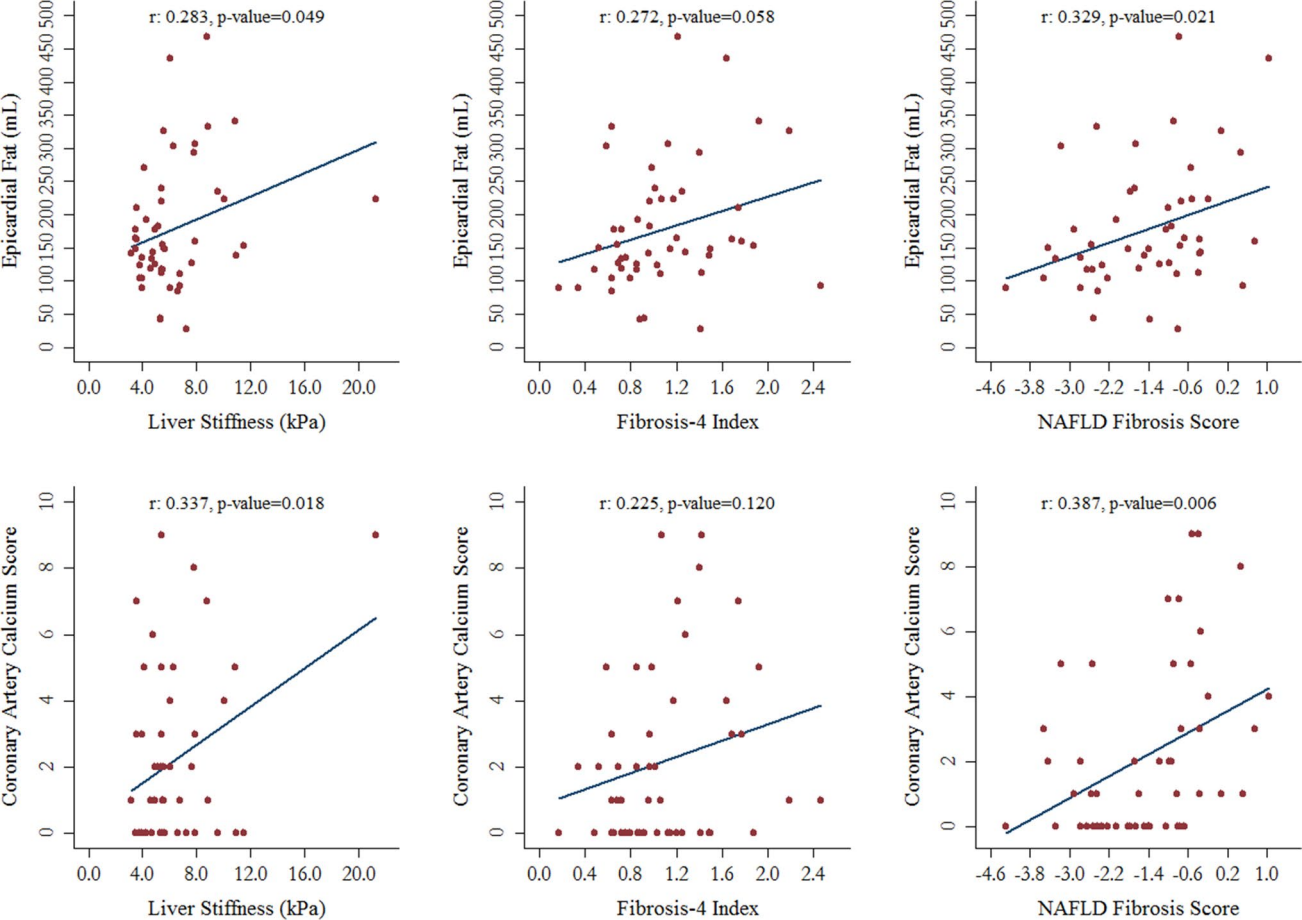
Correlations of liver stiffness and fibrosis serum markers with epicardial fat and coronary artery calcium levels.

Liver stiffness showed a direct association with both EAT (r = 0.283, p-value = 0.049) and CAC levels (r = 0.337, p-value = 0.018) (Fig. 2). The level of liver fibrosis measured by the NFS was moderately associated with EAT (r = 0.329, p-value = 0.021) and CAC (r = 0.387, p-value = 0.006) (Fig. 2). The correlations between FIB-4 Score and EAT or CAC were direct but relatively weak and not statistically significant (Fig. 2).

The identified associations between liver stiffness and EAT remained largely unchanged after adjusting for sex, carbohydrate intolerance/diabetes, obstructive sleep apnea syndrome, hyperuricemia, and obesity in the multivariable regression models (Table 2). Likewise, NFS relationship with EAT remained after adjusting for sex, carbohydrate intolerance, hypertension, hyperuricemia, statin therapy, aspirin therapy, obesity, smoking and VAT/SCAT ratio as independent predictor variables (Table 2).

**Table 2:** Multiple regression analysis with epicardial adipose tissue as the outcome variable.

Model	Liver stiffness (kPa)			Fibrosis-4 Index			NAFLD Fibrosis Score		
	Regression coefficient	SE	p value	Regression coefficient	SE	p value	Regression coefficient	SE	p value
Unadjusted	8.767	4.342	0.049	53.805	27.719	0.058	25.819	10.815	0.021
1	8.719	4.258	0.046	47.390	27.845	0.096	25.188	10.629	0.022
2	9.222	4.435	0.043	59.920	28.850	0.043	25.763	10.974	0.023
3	8.730	4.397	0.053	54.709	28.652	0.062	27.294	11.401	0.021
4	7.976	4.441	0.079	53.526	27.542	0.058	23.771	11.268	0.040
5	6.845	4.147	0.106	41.906	26.421	0.120	18.752	10.702	0.086
6	8.642	4.151	0.043	49.594	26.720	0.070	21.557	10.777	0.051
7	8.305	4.109	0.049	45.050	26.717	0.099	23.946	10.277	0.024
8	8.704	4.325	0.050	53.989	27.588	0.056	24.328	11.001	0.032
9	8.735	4.358	0.051	51.416	28.340	0.076	25.375	11.458	0.032
10	8.816	4.346	0.048	77.369	27.545	0.007	27.409	10.833	0.015
11	9.045	4.573	0.054	53.191	28.681	0.070	25.942	11.233	0.026
12	− 3.754	3.820	0.334	45.719	26.709	0.098	11.258	10.786	0.305
13	6.068	4.642	0.201	67.333	37.009	0.079	30.679	12.711	0.022
14	7.361	4.247	0.090	57.001	26.269	0.035	19.652	11.203	0.086
15	8.575	4.249	0.050	52.207	30.636	0.096	16.009	13.117	0.229

*SE* standard error. Model 1: adjusted for sex. Model 2: adjusted for carbohydrate intolerance. Model 3: adjusted for diabetes. Model 4: adjusted for hypertension. Model 5: adjusted for dyslipidaemia. Model 6: adjusted for obstructive sleep apnoea syndrome. Model 7: adjusted for hyperuricemia. Model 8: adjusted for statin therapy. Model 9: adjusted for aspirin therapy. Model 10: adjusted for obesity. Model 11: adjusted for smoking. Model 12: adjusted for visceral adipose tissue. Model 13: adjusted for visceral adipose tissue/subcutaneous adipose tissue ratio. Model 14: adjusted for presence of carbohydrate intolerance, hypertension, dyslipidaemia, and obesity (number of factors). Model 15: adjusted for presence of carbohydrate intolerance/diabetes, hypertension, dyslipidaemia, and obesity (number of factors).

The relationship between liver stiffness and CAC was preserved even after adjustment for sex, obstructive sleep apnea syndrome, hyperuricemia, statin therapy, aspirin therapy, smoking, and metabolic syndrome features, such as VAT, VAT/SCAT ratio, carbohydrate intolerance/diabetes, hypertension, dyslipidemia, and obesity (Table 3). Similarly, the association between NFS and CAC remained statistically significant after adjustment for sex, obstructive sleep apnea syndrome, hyperuricemia, statin therapy, aspirin therapy, smoking, carbohydrate intolerance/diabetes, hypertension, dyslipidemia, obesity, excepting for VAT (p = 0.263) and VAT/SCAT ratio (p = 0.054). In the number of factors multiple regression analysis, the p-value for the LE association with CAC was 0.017 and for the NFS association with CAC was 0.094.

**Table 3:** Multiple regression analysis with coronary artery score as the outcome variable.

Model	Liver stiffness (kPa)			Fibrosis-4 Index			NAFLD Fibrosis Score		
	Regression coefficient	SE	p value	Regression coefficient	SE	p value	Regression coefficient	SE	p value
Unadjusted	0.289	0.118	0.018	1.227	0.775	0.120	0.840	0.292	0.006
1	0.288	0.116	0.017	1.063	0.782	0.180	0.824	0.288	0.006
2	0.286	0.121	0.022	1.194	0.812	0.148	0.862	0.294	0.005
3	0.276	0.115	0.021	0.963	0.779	0.223	0.733	0.303	0.020
4	0.230	0.113	0.048	1.209	0.715	0.097	0.657	0.288	0.027
5	0.202	0.095	0.040	0.670	0.626	0.290	0.505	0.247	0.047
6	0.285	0.108	0.011	1.076	0.721	0.143	0.687	0.282	0.019
7	0.287	0.119	0.020	1.196	0.791	0.137	0.833	0.295	0.007
8	0.284	0.103	0.008	1.241	0.682	0.075	0.688	0.266	0.013
9	0.286	0.108	0.011	0.886	0.736	0.235	0.636	0.292	0.035
10	0.308	0.122	0.015	1.603	0.822	0.057	0.921	0.302	0.004
11	0.255	0.122	0.043	1.353	0.774	0.087	0.842	0.293	0.006
12	0.282	0.118	0.024	− 0.076	0.934	0.936	0.409	0.358	0.263
13	0.372	0.107	0.002	0.281	1.040	0.789	0.698	0.348	0.054
14	0.241	0.112	0.036	1.334	0.712	0.067	0.626	0.296	0.040
15	0.264	0.106	0.017	1.574	0.774	0.048	0.566	0.330	0.094

*SE* standard error. Model 1: adjusted for sex. Model 2: adjusted for carbohydrate intolerance. Model 3: adjusted for diabetes. Model 4: adjusted for hypertension. Model 5: adjusted for dyslipidaemia. Model 6: adjusted for obstructive sleep apnea syndrome. Model 7: adjusted for hyperuricemia. Model 8: adjusted for statin therapy. Model 9: adjusted for aspirin therapy. Model 10: adjusted for obesity. Model 11: adjusted for smoking. Model 12: adjusted for visceral adipose tissue. Model 13: adjusted for visceral adipose tissue/subcutaneous adipose tissue ratio. Model 14: adjusted for presence of carbohydrate intolerance, hypertension, dyslipidaemia, and obesity (number of factors). Model 15: adjusted for presence of carbohydrate intolerance/diabetes, hypertension, dyslipidaemia, and obesity (number of factors).

## Discussion

The existence of liver fibrosis in NAFLD is strongly associated with an increased risk of major adverse cardiovascular events (MACE)^22,22^, however, it is a challenge to identify subclinical CVD in the highly prevalent NAFLD population. We observed an association of liver stiffness and fibrosis serum markers with subclinical CVD (evaluated through EAT and visual assessment of CAC) in a population of NAFLD patients without personal history of CVD. The novelty of our findings is that fibrosis markers, even at indeterminant range, associate with EAT and CAC. Therefore, our findings support fibrosis screening in NAFLD patients through LE or non-invasive fibrosis markers in clinicians’ daily practice, though they may be useful tools to identify patients with early or subclinical cardiovascular abnormalities.

Cross-sectional and longitudinal studies have found an association between CAC and NAFLD. In 2016, a retrospective study (n = 4731 patients) was conducted to assess the association between NAFLD (diagnosed by ultrasound) and the progression of coronary atherosclerosis (assessed by CT-TS)^8^. The mean duration of follow-up was 3.9 years. The annual rate of progression of coronary calcium was higher in patients with NAFLD (22%) vs. without NAFLD (17%) regardless of cardiovascular and metabolic risk factors (p = < 0.001). Subsequently, a similar cross-sectional study (n = 2345 patients) confirmed the association between NAFLD and CAC (crude OR: 1.631, 95% CI: 1.295–2.053, adjusted OR: 1.348, 95% CI: 1.030–1.765)^9^. Moreover, Lee et al.^24^ found that NAFLD was more closely associated with CAC than abdominal obesity (assessed by waist-hip ratio) after analyzing data from 21,335 male participants. Regarding the presence of fibrosis, our findings are consistent with Song et al. who recently found that non-invasive fibrosis markers (NFS and FIB-4 score) were associated with coronary atherosclerosis measured by Agatston Score (n = 665 NAFLD patients)^25^. We assessed CAC score through visual assessment, which can be easily measured in non-gated non-contrast CT scans, shows good inter-reader correlation, and has an excellent acceptability for non-cardiac radiologists^26^.

Moreover, we simultaneously evaluated EAT which is considered an earlier subrogate marker of CVD, mediator of cardiac arrhythmias^12^ and left ventricular diastolic dysfunction^13^. In 2013, Mahabadi et al.^27^ determine that EAT predicted MACE in the general population in participants from the prospective population-based Heinz Nixdorf Recall cohort (n = 4093 participants, age 59.4 years, 47% male) during a follow-up period of 8.0 ± 1.5 years. Doubling of EAT was associated with a 1.5-fold risk of coronary events when adjusting for cardiovascular risk factors (CVRF) (HR: 1.54; 95% CI: 1.09–2.19), which remained unaltered after further adjustment for CAC (HR: 1.50; 95% CI: 1.07–2.11). Ito et al.^28^, described an association of EAT with an obstructive plaque in patients with zero CAC (n = 1308). EAT may represent an earlier and improved marker of subclinical cardiovascular risk in asymptomatic patients who, therefore, require intensive CVRF treatment. Concerning NAFLD and EAT, in 2014, Petta et al.^10^ found a higher EAT (assessed by echocardiography) in 147 NAFLD patients with histological severe fibrosis vs. mild fibrosis (8.5 ± 3.0 vs. 7.2 ± 2.3 mm; p = 0.006) even after adjusting for gender, age, prediabetes/diabetes, visceral obesity, NASH, and severe steatosis (OR: 1.22; 95% CI: 1.01–1.47; p = 0.04). Authors concluded that EAT is an independent indicator of the severity of liver fibrosis. Similarly, in 2018, EAT (measured by CT-TS) was significantly associated with NAFLD even after adjusting for main CVRF (OR: 1.407; 95% CI: 1.117–1.773) in a 2238 Chinese cohort^29^. In the CAESAR (CArdiometabolic risk, Epicardial fat, and Subclinical Atherosclerosis Registry) (n = 2277 patients) registry, both, EAT (assessed by echocardiography) and the presence of NAFLD were associated with CAC, finding a stronger association to CAC with the presence of increased EAT in NAFLD patients^11^. Interestingly, in a recent prospective, cross-sectional study (n = 100 patients with diabetes)^30^, EAT (measured by magnetic resonance [MR]) was higher in patients with NAFLD. Liver fat content and liver fibrosis (measured by MR elastography) were positively and independently associated with EAT. Authors concluded that liver fat content and fibrosis may increase cardiovascular risk, and therefore, identification of even mild forms of NAFLD in individuals with diabetes may warrant treatment and aggressive risk factor modification to reduce cardiovascular risk. A recent meta-analysis^31^ including thirteen studies (n = 2260 patients) confirmed that EAT was significantly increased in NAFLD patients, it correlated with the severity of hepatic steatosis and with fibrosis.

The underlying mechanisms linking NAFLD and CVD are complex. Both entities share common risk factors, nevertheless, NAFLD seems to be an independent risk factor for CVD^1,1,1,1^. In NAFLD, hepatic fat accumulation results from lipolysis of adipose tissue (AT), dietary fats and de novo lipogenesis with insufficient compensatory fatty acid β-oxidation^6^. This disbalance leads to plasma lipoprotein abnormalities characterized by atherogenic dyslipidemia, which results in fatty acids accumulation and oxidation within the subendothelial vascular wall contributing to endothelial dysfunction^7^ and, subsequent atherosclerosis^33^. Apolipoprotein-B lipoproteins may activate toll-like receptors, and, consequently, innate immune system contributing to systemic inflammation^34^. Additionally, in obesity, adipocytes suffer hypertrophy and hyperplasia with subsequent hypoxia, along with an increase in the number of inflammatory cells that leads to a downregulation of adiponectin expression and secretion of pro-inflammatory factors through activation of metabolic signaling pathways. The concentrations of inflammatory cytokines in the different phenotypes of obesity^35^, supports the continuum of AT dysfunction involved in the onset of systemic low-grade inflammation leading to oxidative stress^34^. The underlying systemic inflammation, in addition, to ectopic dysfunctional fatty tissue accumulation enhances insulin resistance (IR) and may lead to β cell dysfunction in the pancreas^34,34^. In the liver, IR results in compensatory hyperinsulinemia which increases the fatty acid uptake, alters triglycerides transportation and reduces β-oxidation^37^. Progressive liver fat accumulation leads to an activation of hepatic macrophages with a broaden intensification of the pro-inflammatory cytokine activity that is associated with oxidative stress-mediated lipotoxicity and gradual accumulation of excess extracellular matrix through hepatic stellate cells activation^7^. Systemic inflammation and oxidative stress support subclinical organ dysfunction and gradually contributes to the development and progression of atherosclerosis^32,32^. Furthermore, the secretion of proinflammatory cytokines from EAT reinforces the prevailing systemic proinflammatory activity, which in turn results in pathological changes of the coronary arteries^10,10^ and/or structural changes of the contiguous myocardium which can eventually lead to the development of cardiac arrhythmias^12^ and left ventricular diastolic dysfunction^13^. In summary, AT dysfunction^39^ and IR^34^ are important mechanisms linking both entities, although, additional mechanisms, like dysbiosis and genetic susceptibilities, may interplay a role in the pathophysiology of NAFLD and CVD^32,32^.

Current guidelines recommend detailed CVRF assessment in patients with NAFLD^41^. Nonetheless, NAFLD is highly prevalent, and clinicians need an accurate and effective CVD assessment tool, regardless of the presence of traditional risk factors. CAC provides predictive evidence beyond that provided by standard CVRF or risk scores in different ethnic groups^42^. Studies have evidenced that simple visual assessment of the CAC on non-gated thorax CT scan accurately predict incident cardiac events in hospital inpatients^43^ and in an outpatient setting^44^. Several studies have evidenced similar prognostic values and a high degree of agreement between simple visual assessment of CAC and Agatston Score CAC assessment^45–47^. In some populations (i.e. COPD), the visual scaling score of CAC was equally accurate as the Agatston score for prevalent CAD and performed better than the Agatston score in predicting incident cardiac events^48^. With current evidence, in 2016, the Society of Cardiovascular Computed Tomography (SCCT) and the Society of Thoracic Radiology made a class I recommendation in their guidelines for routine CAC visual assessment on non-gated chest CT scans regardless of the indication for the scan^49^. In 2018, the SCCT published the CAC Data and Reporting System (CAC-DRS) to create a standardized method to communicate findings of CAC using traditional Agatston score categories and novel visually estimated CAC^50^. In 2020, the British Society of Cardiovascular Imaging, British Society of Cardiac Computed Tomography, and British Society of Thoracic Imaging recommended the visual quantification of CAC in their consensus statement^51^.

With strong evidence of CVD as a major comorbidity in NAFLD, the use of a simple visual assessment of CAC in readily available images may help to identify patients who may benefit from aggressive CVRF modification in a primary prevention setting. We consider that CAC assessment through visual assessment may be implemented in daily practice for CVD detailed assessment in patients with NAFLD and potential fibrosis assessed through LE and fibrosis markers. Likewise, as current guidelines recommend, our study highlight the importance of NAFLD screening in persons at high CVD risk not only to identify advanced stages of liver fibrosis but also to open therapeutic possibilities that may control CVRF and prevent CVD.

Finally, it is of interest to investigate if the improvement of NAFLD severity may possibly reduce CVRF. A reduction of carotid intima media thickness has been evidenced as NAFLD severity improves^52^. Physicians need to be aware of surveillance and early intervention in NAFLD (weight loss, cardiovascular protection, insulin sensitization and lipid reduction) as the strategy to improve cardiovascular and liver outcomes.

Our findings are relevant in clinical practice as it throws light in the mechanism underlying the association of NAFLD severity with subclinical CVD and suggest potential approaches for CVD prevention. Nevertheless, our study has various limitations: first, it is limited by the retrospective design and a relatively small sample size, however, this exploratory study has a clinically well-characterized cohort with suitable evaluation of subclinical CVD and may be useful to warrant further research. Second, our results derived from middle-aged Spanish adults, though it should be interpreted with caution when applied to different populations. Larger studies are needed to clarify the relationship between liver stiffness and non-invasive fibrosis markers with subclinical CVD. Further research should investigate whether reversal of hepatic steatosis/fibrosis is accompanied by a comparable decrease in overall cardiovascular risk and whether subclinical cardiovascular disease evaluation (through EAT and CAC visual scale) is a cost-effective measure, at least for patients with potential liver fibrosis, independently of the existence of other cardiovascular risk factors.

## Conclusions

LE and noninvasive serum fibrosis markers may be useful tools for identifying NAFLD patients at risk for cardiac abnormalities. The findings of this study suggest direct associations between LE or noninvasive serum liver fibrosis marker, specifically NFS, with EAT and CAC in patients with NAFLD. The association between LE and CAC remained even after adjusting for metabolic syndrome features (carbohydrate intolerance, diabetes, hypertension, dyslipidaemia, visceral fat, and obesity). Our results highlight the importance of assessing the severity of NAFLD not just to determine potential fibrosis but also to aid clinicians in the task of reducing CVRF. In NAFLD, subclinical CVD assessment, through CAC visual scoring, may be a useful tool in patients with potential liver fibrosis, independently of the existence of other CVRF.

## Data Availability

The datasets generated and analyzed during the current study are not publicly available due to containing information that could compromise the privacy of research participants but are available from the corresponding author on reasonable request.
